# Genotyping-by-Sequencing and Morphology Revealed the Role of Polyploidization and Hybridization in the Diversification of the *Centaurea aspera* L. Complex of Section *Seridia* (Juss.) DC. (Asteraceae)

**DOI:** 10.3390/plants11151919

**Published:** 2022-07-25

**Authors:** Hugo Merle, Alfonso Garmendia, María Ferriol

**Affiliations:** 1 Departamento Ecosistemas Agroforestales, Universitat Politècnica de València, 46022 Valencia, Spain; humerfa@upvnet.upv.es; 2 Instituto Agroforestal Mediterráneo (IAM), Universitat Politècnica de València, 46022 Valencia, Spain; algarsal@upvnet.upv.es

**Keywords:** allopolyploidy, autopolyploidy, biogeography, *Centaurea*, Genotyping by Sequencing, hybridization, morphological characterization, section *Seridia*

## Abstract

Hybridization and polyploidy are major drivers of plant evolution. In *Centaurea* (Asteraceae), both mechanisms are frequent and lead to reticulate evolutions. However, in the Western Mediterranean section, *Seridia* studies are scarce. In this section, *Centaurea aspera* forms a complex including four European diploid and one Moroccan autotetraploid subspecies, an allopolyploid, and hybrids among them. Here, we aimed to delimit the different taxa, identify any introgressions, and discuss their evolutionary history. Samples of all taxa were analysed using 1688 SNPs obtained through GBS and were morphologically characterized. Three genetically well-differentiated clusters were observed, corresponding to the allopolyploid *C. seridis*, the diploid *C. aspera* and the cryptic autotetraploid *C. aspera* ssp. *gentilii*, which is proposed to be considered as a species. *Centaurea seridis* showed a high isolation by distance, a greater morphological variability, and a lack of interspecific gene flow. Diploid and autotetraploid *C. aspera* individuals were morphologically similar, and some introgressions were detected in Southern Spain, where new forms may promote diversification. This gene flow might have taken place during the Messinian and before autopolyploidization occurred in Morocco. In the *C. aspera* complex, current interspecific barriers are strong, while polyploidization may provide a better adaptation to drier environments.

## 1. Introduction

Interspecific hybridization is considered to be an important mechanism in angiosperm speciation and evolution with several consequences, such as introgressions [1], extinction and displacement of parental species [2] and formation of new stable lineages [3]. Besides hybridization, it is now widely recognized that polyploidy has been playing an extremely important role in plant evolution, although its significance has long been debated [4]. Both mechanisms can occur in nature as isolated or combined. Hybridization can occur between species or cytotypes of the same ploidy level (homoploid hybridization) or of different ploidy levels (heteroploid hybridization) [3]. However, offspring frequently results in sterile hybrids because of pairing disequilibrium at meiosis. Although sterility mostly increases in heteroploid hybrids because they have an odd chromosome basic number and act as a triploid block, they can sometimes result in viable offspring because of the production of ploidy variable gametes, acting by contrast as a triploid bridge [5]. Additionally, hybridization may be associated with polyploidization, producing allopolyploids and restoring fertility [6]. Allopolyploids are widely assumed to have a higher plasticity and a better adaptation to changing environments than their diploid relatives because they benefit not only from parental phenotypic expressions patterns but also from epigenetic changes, new functionalizations and varying expression levels of duplicated genes and frequent subsequent diploidizations [7]. By contrast, autopolyploidy, formed through genome duplication within a single species, was classically considered as rare and maladaptive. However, recent research suggests the contrary: autopolyploids have a much higher incidence in nature than previously thought and may also benefit from increased genetic variability and adaptability [8]. Both allopolyploidy and autopolyploidy are major players in evolution because they can lead to instant speciation in one or two generations [3,9]. In addition, most allopolyploids and autopolyploids may have arisen recurrently from separate hybridization events [8].

In nature, both hybridization and polyploidization act in complex and frequently long processes (i.e., [10]), which may result in a reticulate pattern of evolution because whole genome duplication can remove the reproductive barriers that arise in homoploid interspecific hybrids [11]. Hybridization and polyploidy have been reported to be especially important drivers of plant diversification in the Mediterranean region because of its complex geological history with successive changes in land connections, climatic oscillations, and human activities that have been developed since ancient times [6]. These authors reported that in the Iberian Peninsula, 12.7% and 48.8% of the cited plants have a hybrid and polyploid origin, respectively. Specifically, the Asteraceae family has been reported to have many hybrid species [12] and 46% of polyploid species, with 55.6% of them having populations with mixed ploidy levels [6].

Within Asteraceae, the genus *Centaurea* L. includes nearly 250 species and is particularly diversified in the Mediterranean Basin and the Irano-Turanian region [13]. This high rate of diversification is partly the result of the existence of cycles of polyploidy, descending dysploidy, and hybridization events [14,15,16]. Within *Centaurea*, three subgenera and twenty-three sections have been described with three main distributions: western Mediterranean, eastern Mediterranean, and circummediterranean and Eurosiberian [13]. Phylogenetic relationships and the influence of hybridization and polyploidization in natural evolution have been well studied in some sections of *Centaurea* [16,17,18,19,20,21,22,23]. For instance, the circummediterranean section *Acrocentron* (Cass.) DC. of subgenus *Lopholoma* (Cass.) Dobrocz. has a complex evolution, with ploidy levels ranging from diploid to endecaploid and several cases of allopolyploidy and autopolyploidy [9,17]. Taxa show little or no barriers against interspecific hybridization, often resulting in homoploid hybrids that favour gene flow. Consequently, high levels of hybridization, introgression and reticulation exist [18,19]. The Mediterranean and Anatolian subgenus *Cyanus* (Mill.) Cass. ex Hayek also showed a still-incomplete evolutionary differentiation of taxa, with the existence of a high degree of hybridization and introgression, along with complex series of dysploidy in annual representatives [20]. In the circummediterranean clade of subgenus *Centaurea* (*Centaurea* group), polyploid taxa represent only ca 13%, and hybridization events have been identified as major drivers of reticulate evolution [21]. The weak reproductive barriers and the high frequency of fertile homoploid hybrids may have caused extensive introgressions [21,22,23,24].

However, other well-differentiated clades in *Centaurea* have been poorly studied, such as the western Mediterranean clade of subgenus *Centaurea*. This is the clade in which natural classification of sections is most difficult. It includes the sections *Seridia* (Juss.) DC. and *Melanoloma* (Cass.) DC., which are found exclusively in the western Mediterranean, together with sections *Hymenocentron* (Cass.) DC. and *Mesocentron* (Cass.) DC., which are made up of widely distributed species [16]. Most species from section *Seridia* are perennial herbs that mainly develop on dunes, places with periodical inundations and coastal rocks [14]. The section includes ca 20 species which are distributed in western Europe, reaching the Atlantic coasts of Portugal and Morocco [25], as well as other north African countries [26]. Taxonomical and caryological studies of this section are scarce and include mainly European and Moroccan species. Only three taxa have been cited as tetraploids: *C. seridis* L., *C. sphaerocephala* L. [27] and *C. aspera* L. ssp. *gentilii* (Braun-Blanq. & Maire) Dobignard [28]. In addition, as related species are not so closed, hybridization usually produces sterile offspring and therefore they do not lead to reticulation [13]. *Centaurea aspera* L. is considered to be the most widespread species of the section, being present in western Mediterranean and introduced in central and north Europe, North America and Australia [25]. It comprises five subspecies, four of which are diploid and distributed in Europe (ssp. *aspera* L., ssp. *stenophylla* (Dufour) Nyman, ssp. *pseudosphaerocephala* (Shuttlew. ex Rouy) Gugler, and ssp. *scorpiurifolia* (Dufour) Nyman) and one is an autotetraploid distributed in the Atlantic coast of Morocco (ssp. *gentilii*) [29]. *Centaurea aspera* is also one of the parentals of the allopolyploid *C. seridis*, with the other parental being an unknown taxon [30]. *Centaurea seridis* develops on the coasts of Mediterranean Spain and the Atlantic and Mediterranean Morocco, and includes three subspecies in its Spanish distribution area (ssp. *sonchifolia* (L.) Greuter, ssp. *maritima* (Dufour) Dostál, and ssp. *cruenta* (Willd.) Dostál) [29] and several varieties in Morocco [31]. In addition, at least in the Iberian Peninsula, all the few homoploid and heteroploid hybrids found in section *Seridia* involve *C. aspera* [25].

Polyploid hybrid complexes derived from *C. aspera* are thus formed in several contact zones. In the Spanish Mediterranean coast, the distributions of *C. seridis* and *C. aspera* ssp. *stenophylla* overlap in few contact zones, and sterile triploid hybrids are produced involving different *C. seridis* subspecies (*C.* x *subdecurrens* Pau nothossp. *subdecurrens* and nothossp. *oblanceolata* Merle, Garmendia & Ferriol derived from *C. seridis* ssp. *maritima* and ssp. *cruenta*, respectively) [30,32]. The distribution areas of the tetraploids *C. seridis* and *C. aspera* ssp. *gentilii* also overlap in a contact zone of Morocco, and sterile tetraploid hybrids arise (*C.* x *subdecurrens* nothossp. *paucispina* Ferriol, Merle & Garmendia) [31]. The mating system of the taxa involved in all these contact zones has been well characterized, and the results show that while diploid and autotetraploid individuals of *C. aspera* are strictly allogamous, the allotetraploid *C. seridis* is highly autogamous [33,34,35]. However, genetic relationships and gene flow have only been studied in Spanish contact zones using microsatellites and other dominant molecular markers. These studies revealed the absence of gene flow between *C. aspera* and *C. seridis* [36], and a higher population structure and isolation by distance in *C. seridis* than in *C. aspera* [30]. However, the joint genetic analysis of all the *C. aspera* and *C. seridis* subspecies and their hybrids has not been performed yet.

In this context, we aimed to deepen in all the known hybrid and polyploidy complexes derived from *C. aspera*, considering all the taxa involved and genetic, morphologic and biogeographic criteria. Genetic analysis was performed using Genotyping By Sequencing (GBS), which allowed for the use of a large array of high-quality single nucleotide polymorphism (SNP) markers. The following questions were addressed: (i) How many and which taxa can be genetically and/or morphologically recognized? (ii) Is there any evidence of hybridization or gene flow among taxa or ploidy levels? (iii) Is there a geographical distribution within or among taxa or ploidy levels that can be related to their evolutionary history?

## 2. Results

### 2.1. STRUCTURE Analysis

**Figure 1:**
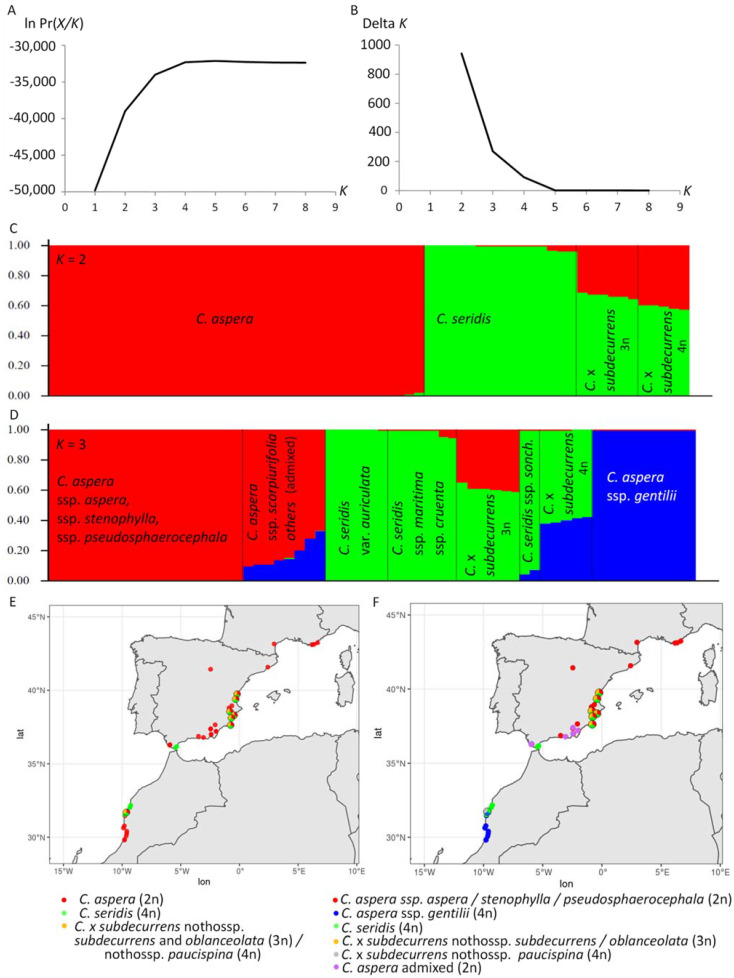
STRUCTURE clustering analysis for *Centaurea aspera*, *C. seridis* and *C.* x *subdecurrens* hybrids between them in the studied area. (**A**) Diagram representing log-likelihood of the SNP data for *Centaurea* individuals given *K* clusters obtained through 10 runs with the algorithm. (**B**) Diagram representing Delta *K* statistics of Evanno et al. [37]. (**C**) Diagram representing alignment tests for number of clusters *K* = 2, and (**D**) *K* = 3, with individual plants represented by columns and taxonomic adscription of individuals indicated. (**E**) Geographic location of STRUCTURE clusters with *K* = 2, and (**F**) *K* = 3. Pie charts are depicted in contact zones. Maps were made with Natural Earth (https://www.naturalearthdata.com/ accessed on 1 June 2022).

Diagrams of the STRUCTURE analysis representing the log-likelihood of the 1688 SNP data and the Delta *K* statistics of Evanno et al. [37] on the 63 *Centaurea* individuals are shown in Figure 1A,B, respectively. Maximum ln Pr (*X*/*K*) was reached at *K* = 2. Hence, individuals of *Centaurea* formed two genetic clusters in the sampled area, corresponding to *C. aspera* and *C. seridis* (red and green colours, respectively, in Figure 1C). As expected, the *C.* x *subdecurrens* hybrids between both species appeared as highly admixed. The triploid hybrids (nothossp. *subdecurrens* and *oblanceolata*) showed an average individual membership coefficient to *C. aspera* and *C. seridis* of 0.33 and 0.67, respectively, while in the tetraploid nothossp. *paucispina*, these coefficients were 0.40 and 0.60, respectively. Two individuals of *C. seridis* ssp. *maritima* and one of ssp. *sonchifolia* appeared to have a low membership coefficient to *C. aspera* (close to 0.04) (Figure 1C). *Centaurea aspera* was widely distributed in Spain and Southern France and was also present south of the sampled locality of Zaouiat el Kourati on the Moroccan Atlantic coast, while *C. seridis* was found on the southern half of the Spanish Mediterranean coast and north of Zaouiat el Kourati on the Moroccan Atlantic coast (Figure 1E). Hybrids between them appeared wherever the distribution areas of *C. aspera* and *C. seridis* contacted.

Furthermore, a second peak was found at *K* = 3 (Figure 1B), suggesting that the 63 plants could be further divided into three clusters, corresponding to the European diploid subspecies of *C. aspera* (ssp. *aspera*, ssp. *stenophylla*, ssp. *scorpiurifolia*, and ssp. *pseudosphaerocephala*), the Moroccan tetraploid *C. aspera* ssp. *gentilii*, and *C. seridis* (red, blue and green colours, respectively, in Figure 1D,F). In this new analysis, the triploid *C.* x *subdecurrens* individuals (nothossp. *subdecurrens* and *oblanceolata*) appeared to be hybrids between diploid *C. aspera* and tetraploid *C. seridis*, while nothossp. *paucispina* individuals appeared to be hybrids between the tetraploid *C. aspera* ssp. *gentilii* and *C. seridis* (Figure 1D). Some other admixed individuals between diploid and tetraploid *C. aspera* were observed only in Andalusia, in southern Spain (Figure 1D,F). All of them were diploid and included all the five Andalusian endemic *C. aspera* ssp. *scorpiurifolia* individuals with an average membership coefficient to ssp. *gentilii* of 0.13, two ssp. *aspera* individuals with an average coefficient of 0.31, and one ssp. *stenophylla* individual with a coefficient of 0.15. In addition, two *C. seridis* ssp. *maritima* showed an average membership coefficient to diploid *C. aspera* close to 0.05, while the two *C. seridis* ssp. *sonchifolia* present in Southern Spain showed an average membership coefficient to *C. aspera* ssp. *gentilii* close to 0.06 (Figure 1D,F).

Finally, although much less intense, a third peak was found at *K* = 4 (Figure 1B). In this new grouping, the diploid *C. aspera* individuals were further divided into two clusters. One cluster (cluster 3) included *C. aspera* ssp. *pseudosphaerocephala* individuals and most individuals of ssp. *aspera* and ssp. *stenophylla*, which were widely distributed in Spain and southern France. The other cluster (cluster 4) included all the *C. aspera* ssp. *scorpiurifolia* and one Andalusian ssp. *stenophylla* individuals with an average membership coefficient of 0.85, and an average membership coefficient to the remaining diploid *C. aspera* of 0.13. Two Andalusian *C. aspera* ssp. *aspera* individuals appeared to be admixed, with average membership coefficients to cluster 3, cluster 1 (*C. aspera* ssp. *gentilii*), and cluster 4 of 0.56, 0.29, and 0.15, respectively (Figure S1). These three *C. aspera* ssp. *stenophylla* and *aspera* individuals were the same that appeared to be admixed at *K* = 3.

### 2.2. Genomic Relationships among Individuals

**Figure 2:**
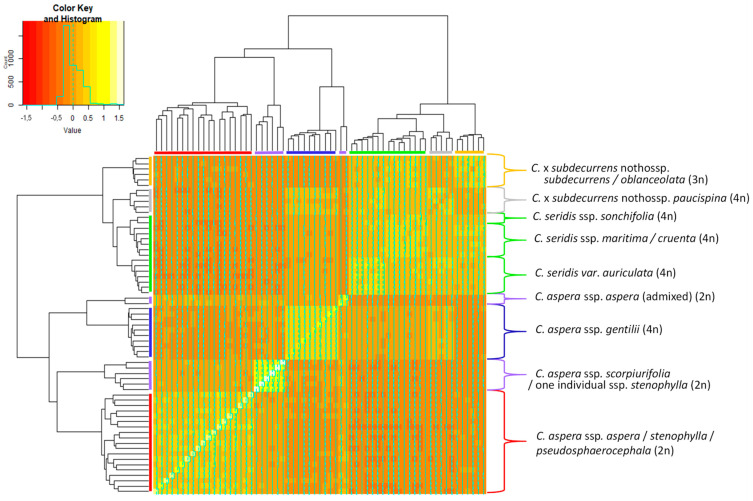
Heatmap depicting genomic relationships between the studied *Centaurea* individuals. Individuals are across the vertical and horizontal axes, with each square indicating the genomic relationship between the two respective individuals. The magnitude of the relationship is indicated by the colour range as shown in the colour key (1 being the relationship between an individual and itself and 0 being the mean of the data set). Colours in the branches of the tree correspond to different populations/taxa.

The heatmap that represents the genomic relationship matrix which estimates the true proportion of the genome shared between individuals following Yang et al. [38], along with a tree based on these genomic relationships, is depicted in Figure 2. The grouping of individuals agreed with the clusters obtained with STRUCTURE. Two main branches in the tree were found, one including the *C. aspera* individuals, and the other the *C. seridis* individuals and the interspecific hybrids between them. Within each branch, individuals were related with lighter colour than between the two branches.

In addition, within the *C. aspera* group, four further clear subgroups were observed. The first subgroup corresponded to the tetraploid *C. aspera* ssp. *gentilii* that was more related to *C. seridis* (lighter colours among individuals) than the other two subgroups (darker colours among individuals). The second subgroup included the diploid *C. aspera* ssp. *scorpiurifolia* individuals and the Andalusian ssp. *stenophylla* individual that was also grouped with ssp. *scorpiurifolia* in the STRUCTURE analysis, both at *K* = 3 and *K* = 4. The third subgroup comprises all the individuals except for three of the diploid *C. aspera* ssp. *aspera*, ssp. *stenophylla*, and ssp. *pseudosphaerocephala*. Finally, the fourth subgroup included two individuals of *C. aspera* ssp. *aspera*, which showed similar proportions of the genome shared with the tetraploid *C. aspera* ssp. *gentilii* and with the diploid *C. aspera* subspecies (similar colours among individuals). These two individuals were the same that appeared as admixed in the STRUCTURE analysis.

Within the *C. seridis* group, the Spanish individuals (ssp. *sonchifolia*, *maritima*, and *cruenta*) and the Moroccan plants (var. *auriculata* (Balb.) Ball) appeared slightly differentiated. The tetraploid hybrid *C.* x *subdecurrens* nothossp. *paucispina* was related to both *C. aspera* ssp. *gentilii* and *C. seridis*, while the triploid hybrid *C.* x *subdecurrens* (including nothossp. *subdecurrens* and *oblanceolata*) was more related to *C. seridis* than to *C. aspera*.

### 2.3. Population Diversity and Differentiation

The total heterozygosity was 0.291. Considering the two clusters obtained with STRUCTURE (*K* = 2), heterozygosity was higher in *C. seridis* than in *C. aspera* (0.231 and 0.192, respectively), with a maximum in the admixed triploid and tetraploid hybrids (0.334) (Table 1). Within *C. aspera* (*K* = 3), heterozygosity of the cluster including diploid ssp. *aspera*, ssp. *stenophylla*, and ssp. *pseudosphaerocephala* was higher than the cluster including autotetraploid ssp. *gentilii* (0.165 and 0.109, respectively). Considering the further grouping within diploid *C. aspera* obtained with STRUCTURE (*K* = 4), cluster 3 including ssp. *pseudosphaerocephala* and most individuals of ssp. *aspera* and ssp. *stenophylla* showed a higher heterozygosity than cluster 4 including ssp. *scorpiurifolia* and three Andalusian ssp. *aspera* and ssp. *stenophylla* individuals (0.144 and 0.088, respectively) (Table S1).

Both G’_ST_ [39] and F_ST_ showed significant and large genetic differentiation among clusters (Table 1 and Table S1). Values of G’_ST_ were 0.315, 0.412, and 0.461 at number of clusters *K* = 2, 3, and 4, respectively, and values of F_ST_ were lower: 0.209, 0.279, and 0.271 at number of clusters *K* = 2, 3, and 4, respectively. In agreement with these results, AMOVA analysis also showed a high differentiation among clusters (Table 2 and Table S1). Considering the two clusters *C. aspera* and *C. seridis* (*K* = 2), 46.01% of the variance was among species and 53.98 within species. Within *C. aspera*, a high differentiation was also found between diploid and tetraploid populations (39.19% of the variance). Finally, within diploid *C. aspera*, 48% of the variance was found between clusters 3 and 4.

### 2.4. Isolation by Distance

**Figure 3:**
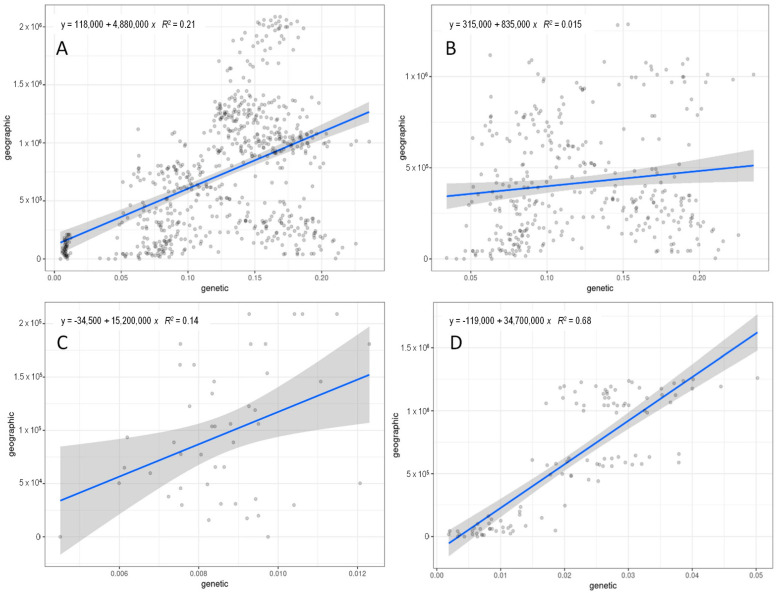
Geographical distances (m) versus genetic distance among locations for: (**A**) the *Centaurea aspera* individuals, (**B**) *C. aspera* diploid individuals (ssp. *aspera*, ssp. *pseudosphaerocephala*, ssp. *stenophylla*), (**C**) *C. aspera* tetraploid individuals (ssp. *gentilii*), and (**D**) tetraploid *C. seridis* individuals. The regression is represented by a blue line. The ribbon shows the 0.95 confidence interval.

Mantel test [40] performed with *C. aspera* individuals, including all the subspecies, resulted in a significant high correlation between genetic and geographic distances (*r* = 0.461, *p* = 0.001) (Figure 3). However, considering only diploid *C. aspera* (ssp. *aspera*, ssp. *pseudosphaerocephala*, ssp. *stenophylla*), a nonsignificant, low correlation was found (*r* = 0.124, *p* = 0.117). In contrast, the tetraploid *C. aspera* ssp. *gentilii* displayed a low but significant isolation by distance (*r* = 0.378, *p* = 0.004), while a significant and high correlation was found among the *C. seridis* locations (*r* = 0.825, *p* = 0.001). These results are in agreement with those obtained with STRUCTURE and the population differentiation estimators, which separated the *C. aspera* Spanish diploid and Moroccan tetraploid individuals, and with genomic relationships, which also showed two subgroups within *C. seridis* corresponding to Spanish and Moroccan populations.

### 2.5. Morphological Characterization

Out of the twenty-nine vegetative traits and the nine reproductive traits evaluated, twenty and five traits respectively showed significant differences among diploid *C. aspera* (ssp. *aspera*, ssp. *pseudosphaerocephala*, ssp. *stenophylla*), tetraploid *C. aspera* ssp. *gentilii*, and tetraploid *C. seridis* (Table 3). *Centaurea seridis* was the most differentiated taxon; it differed from *C. aspera* in all the morphological traits that turned out to be significant except for the internode length between medium leaves. The *C. aspera* diploid individuals were similar to the *C. aspera* ssp. *gentilii* tetraploid individuals. Tetraploid individuals differed from diploids only in their higher thickness of upper and medium leaves and shorter internode length between medium leaves. As expected, both triploid and tetraploid *C.* x *subdecurrens* showed intermediate values between those of parentals in most of the morphological traits.

**Figure 4:**
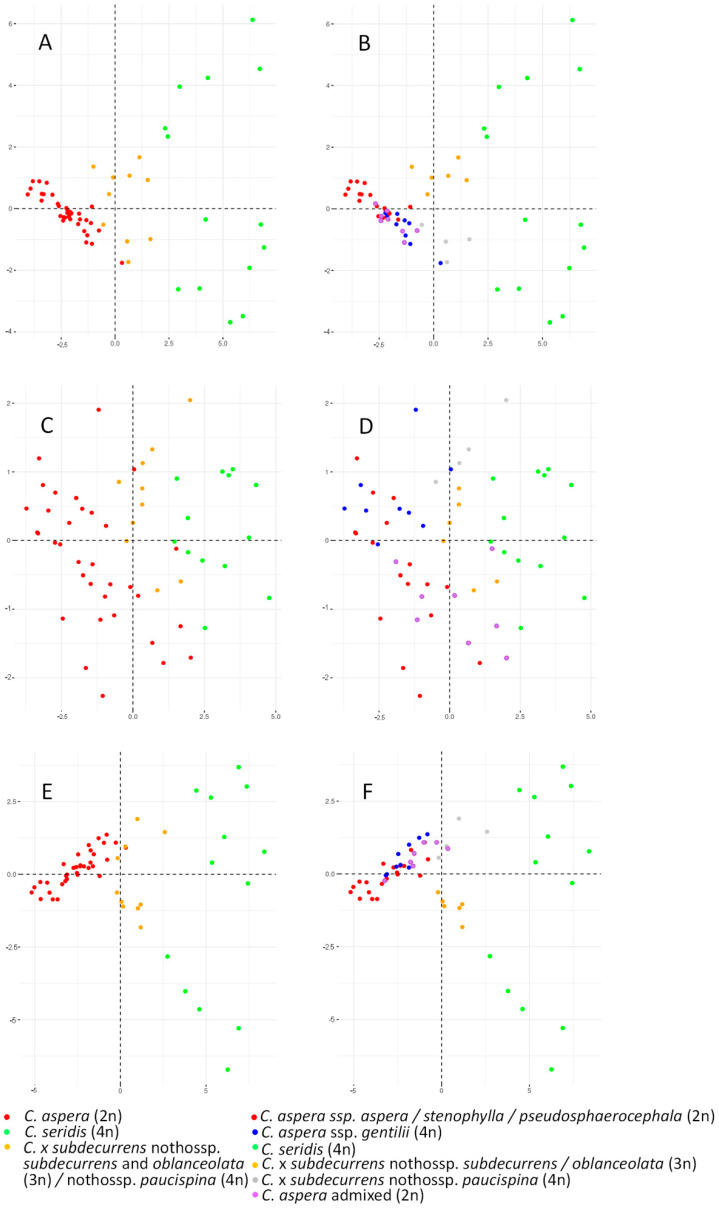
Two-dimensional plots of the principal component analyses based on the morphology of the *Centaurea aspera*, *C. seridis* and *C.* x *subdecurrens* hybrid individuals. STRUCTURE analysis was used for establishing genetic populations. (**A**) Vegetative characters at *K* = 2 and (**B**) at *K* = 3. (**C**) Reproductive characters at *K* = 2 and (**D**) at *K* = 3. (**E**) Vegetative and reproductive characters at *K* = 2 and (**F**) at *K* = 3.

The PCA performed on the *Centaurea* individuals using vegetative, reproductive, and both characters at once are shown in Figure 4. The first principal component accounted for 50.3%, 71.4%, and 51.7% of the total variance for the vegetative, reproductive, and all characters, respectively, and the second principal component for 15.8%, 12.7% and 12.7% of the total variance, respectively. Considering the two clusters obtained with STRUCTURE (*C. aspera* and *C. seridis*), the vegetative characters and all the characters at once clearly differentiated both species, with the *C.* x *subdecurrens* hybrids appearing as intermediate between them. While *C. aspera* individuals showed a high vegetative uniformity, those of *C. seridis* were more variable according to the high dispersion in the second component. The differentiation between the two species was less clear using reproductive characters alone, and a certain overlap was observed. In this case, both *C. aspera* and *C. seridis* showed a high variability. When the genetic differentiation between the diploid *C. aspera* (ssp. *aspera*, *stenophylla* and *pseudosphaerocephala*) and the tetraploid ssp. *gentilii* was also considered (*K* = 3 clusters obtained with STRUCTURE), only a slight differentiation and a great overlap between the diploid and tetraploid *C. aspera* individuals was observed with all the character types. In relation to hybrids, the triploid and tetraploid *C.* x *subdecurrens* were differentiated particularly when using vegetative characters and all the characters at once, although this differentiation was not so clear when using only reproductive characters. Finally, considering four clusters obtained with STRUCTURE, a morphological continuum was observed among clusters within *C. aspera* (diploid ssp. *aspera*, *stenophylla*, and *pseudosphaerocephala*; diploid ssp. *scorpiurifolia* and *stenophylla*; tetraploid ssp. *gentilii*; and admixed individuals between clusters) (Figure S2). However, diploid ssp. *scorpiurifolia* and admixed individuals were certainly more similar to ssp. *gentilii* when using vegetative characters but were more similar to the remainder of diploid subspecies when using reproductive characters.

### 2.6. Comparison of Genetic and Morphologic Relationships

**Figure 5:**
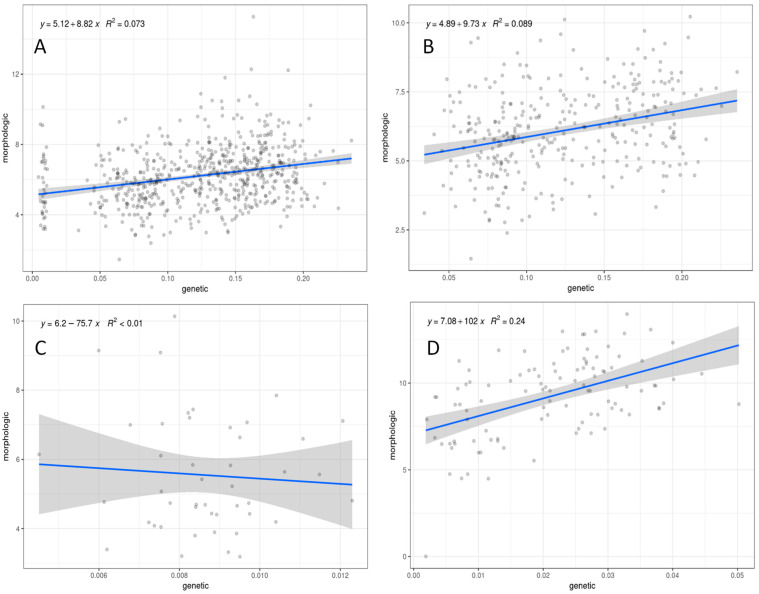
Euclidean distances using standardized morphological variables versus genetic distance for: (**A**) the *Centaurea aspera* individuals, (**B**) *C. aspera* diploid individuals (ssp. *aspera*, ssp. *pseudosphaerocephala*, ssp. *stenophylla*), (**C**) *C. aspera* tetraploid individuals (ssp. *gentilii*), and (**D**) tetraploid *C. seridis* individuals. The regression is represented by a blue line. The ribbon shows the 0.95 confidence interval.

Mantel test performed with *C. aspera*, including all the subspecies, resulted in a significant correlation (*r* = 0.271, *p* = 0.001) (Figure 5). Similarly, considering only diploid *C. aspera* (ssp. *aspera*, ssp. *pseudosphaerocephala*, ssp. *stenophylla*), a significant correlation was also found (*r* = 0.299, *p* = 0.001). By contrast, the correlation of the Mantel test including only the tetraploid *C. aspera* ssp. *gentilii* was nonsignificant (*r* = −0.157, *p* = 0.518). In relation to *C. seridis*, a significant and high correlation was found between genetic and morphological distances (*r* = 0.488, *p* = 0.001).

## 3. Discussion

### 3.1. Genetical and Morphological Differentiation of Taxa and Gene Flow among Them

The diploid *Centaurea aspera* and the tetraploid *C. seridis* appeared clearly differentiated both morphologically and genetically. In addition, despite being highly autogamous [33,35], *C. seridis* displayed a high heterozygosity, strongly suggesting an allopolyploid origin, in agreement with the high fixed heterozygosity observed using microsatellites [30]. Within *C. aspera*, two clear genetic groupings were observed that showed differentiated geographic distributions and ploidy levels: European diploid individuals of ssp. *aspera*, *stenophylla*, *pseudosphaerocephala* and *scorpiurifolia* and Moroccan tetraploid individuals of ssp. *gentilii*. However, individuals of these two genetic clusters could not be differentiated by means of morphology alone. This lack of consistent morphological differences points to an autopolyploid origin of *C. aspera* ssp. *gentilii*, as occurring with other *Centaurea* autopolyploids (i.e., *C. stoebe* L. [41], *C. toletana* Boiss. & Reut. [10], and *C. phrygia* L. [42]). An autopolyploid origin is here also supported by a heterozygosity that is even lower than that displayed by the diploid representatives of *C. aspera* and that may be due to bottleneck effects related to autopolyploidization events. However, strong genetic differentiation between both taxa were observed, and previous studies showed that artificial pollinations between diploid *C. aspera* ssp. *stenophylla* and tetraploid ssp. *gentilii* produced seeds in both directions which aborted half of the times, and those that were viable were mostly triploids, supporting strong postzygotic barriers [35]. The presence of these interspecific reproductive barriers may lead to independent evolutions and a clear genetic differentiation of diploids and tetraploids [23], supporting the consideration of *C. aspera* ssp. *gentilii* as a species (*C. gentilii* Braun-Blanq. & Maire) rather than as a subspecies. This change in rank is aligned with the suggestions of Soltis et al. [8] and Levin [9], who stated that autopolyploidization is a significant mechanism of speciation and that many autopolyploids have gone undetected and may represent cryptic species. The consideration of *C. aspera* ssp. *gentilii* as a species was also proposed by Ferrer-Gallego et al. [43], who typified the name based on the original protologue of *C. gentilii* that included a synonym (*Centaurea fragilis* Durieu var. *integrifolia* Ball), a complete description, a diagnosis against *C. fragilis*, and some comments on its ecology and distribution in Morocco [44], and designated the lectotype for *C. gentilii*. As a consequence, we also propose a change in rank of the tetraploid hybrids, from *C.* x *subdecurrens* nothossp. *paucispina* to *C.* x *paucispina*, which was also suggested by Ferrer-Gallego et al. [45].

The hybrid genetic position of triploid *C.* x *subdecurrens* individuals (including nothossp. *subdecurrens* and nothossp. *oblanceolata*) between diploid *C. aspera* and tetraploid *C. seridis* was confirmed [36], and that of tetraploid nothossp. *paucispina* between tetraploid *C. aspera* ssp. *gentilii* and *C. seridis* was assessed here for the first time. Although diploid *C. aspera* and allopolyploid *C. seridis* are able to hybridize in natural contact zones [30], SNPs analysis in STRUCTURE resulted in a segregation of one *C. aspera* to two *C. seridis* in the triploid hybrids, which strongly support that all the hybrids represent true F1 offspring and are completely sterile, as has previously been shown using artificial pollinations [33]. The segregation of tetraploid hybrids between *C. aspera* ssp *gentilii* and *C. seridis* was two ssp. *gentilii* to three *C. seridis*. Although this result suggests some introgressions from *C. seridis* to ssp. *gentilii*, these were not evident using STRUCTURE in any of the individuals of ssp. *gentilii* sampled. Furthermore, in the natural contact zone, we could not find any cypsela on *C.* x *subdecurrens* nothossp. *paucispina* [31]. One possible explanation is that *C. aspera* ssp. *gentilii* has more SNP alleles in common with *C. seridis* than the diploid *C. aspera* subspecies, as supported by the genomic relationships, leading to a higher representation of *C. seridis* in the genome of *C. subdecurrens* nothossp. *paucispina*.

In relation to the diploid subspecies of *C. aspera*, Andalusian ssp. *scorpiurifolia* appeared to be the most genetically differentiated. Morphologically, ssp. *scorpiurifolia* individuals showed vegetative characteristics more similar to those displayed by ssp. *gentilii* and reproductive characters more similar to those displayed by ssp. *aspera*, ssp. *stenophylla* and ssp. *pseudosphaerocephala*, although a continuum exists as previously found in *C. aspera* individuals growing in Andalusia [46]. One ssp. *stenophylla* individual appeared to be related to ssp. *scorpiurifolia*, which can be due to a wrong adscription given this morphological continuum. However, in contrast with the high sterility of all the *C.* x *subdecurrens* hybrids, STRUCTURE analysis showed that the diploid ssp. *scorpiurifolia* individuals and two more Andalusian ssp. *aspera* individuals appeared clearly admixed between diploid *C. aspera* and tetraploid *C. aspera* ssp. *gentilii*. The possibility of hybridization between diploid and tetraploid *C. aspera* was also observed when we performed artificial pollinations and obtained 1.08% of the viable seeds which were tetraploid, with the remaining triploids having no diploid representatives [35]. These results agree with the review of Schmickl et al. [47], who reported that interploidal introgression generally occurs unidirectionally, from diploids to polyploids, although there is evidence that occasionally it can also occur in the reverse direction. Consequently, the observed introgressions may be more related to the historical biogeography of taxa as is discussed below.

### 3.2. Geographic Distribution of Taxa and Evolutionary History

Results of the study supported that *C. aspera*, with a high genetic diversity and the widest distribution area of all the section *Seridia* taxa [25], is able to originate polyploids and hybrids with many *Centaurea* taxa that even belong to sections different than *Seridia*, such as *Centaurea pullata* L. (section *Melanoloma*) [48], *C. calcitrapa* M.Bieb. (section *Calcitrapa* DC.), [49], or *C. resupinata* Coss. ssp. *saguntina* (Mateo & M.B.Crespo) Greuter (section *Centaurea* subsection *Willkommia* Blanca) [50].

Although only diploid *C. aspera* individuals were present in Europe and only tetraploid individuals in Morocco, gene flow has existed between them in southern Spain as supported by STRUCTURE analysis. Recent gene flow is unlikely because interploidal introgressions from polyploids to diploids is highly infrequent [47] and because pollen and seed dispersal in *Centaurea* is fairly limited [51,52,53]. A more likely hypothesis is that diploid *C. aspera* individuals already existed during the late Miocene, which has also been reported to be the period when many *Centaurea* clades originated and diversified. For instance, the circummediterranean *Centaurea* group arose in Eastern Mediterranean ca. 8.4 Mya. [21], section *Cyanus* originated ca. 6–7 million years ago [20], and section *Centaurea* from Sardinia started to diversify between approximately 11.1 and 5.1 Mya [23]. Individuals related to ssp. *scorpiurifolia* may have been distributed in the Southern Iberian Peninsula and Morocco during the Messinian Salinity Crisis (between 6 and 5.3 Mya), when both lands were connected due to subduction processes in the westernmost Mediterranean, causing the closure of the marine gateways that existed between the Atlantic Ocean and the Mediterranean Sea [54]. Evidences of migration of several *Centaurea* species between the Iberian Peninsula and Northern Africa during the Messinian Salinity Crisis are also reported for the circummediterranean *Centaurea* group [21] and section *Acrocentron* of subgenus *Lopholoma* [19]. In the western Mediterranean, a southward increase in herbs, especially subdesertic, and a decrease in pines and other trees has been reported from the Messinian to Lower Pliocene, evidencing that the environment was drier and more open from Catalonia (Spain) until Rabat (Morocco) [55]. Furthermore, especially in the area of the Strait of Gibraltar, a great anthropogenic impact including fires and herding occurred after the Messinian Salinity Crisis [56]. These high environmental stresses in Morocco may have potentiated the emergence of ssp. *gentilii* after the opening of the Strait of Gibraltar. It has been shown that production of unreduced gametes and autopolyploidization events are stimulated by environmental stresses in nature [57]. Because polyploids may generate new genetic diversity, they may have been under positive selection associated with an increased adaptability and ecological tolerance of individuals [58]. A shift from diploid to tetraploid is often associated with increased tolerance to drought, salt, temperature fluctuations and high levels of herbivory [9]. Along with the formation of autopolyploids, a genetic divergence of populations in both sides of the Strait of Gibraltar, which is considered to be a greater biogeographic barrier than the Pyrenees or the Alps, may have occurred, although the effectiveness of the Strait to allow or interrupt genetic exchange between Africa and Europe depends on the plant group and is not related to dispersal abilities [54]. In *Centaurea*, genetic differentiation between the western Mediterranean and Moroccan populations has effectively been observed in subsection *Willkommia* of section *Centaurea*, showing a split of ITS and 3′ETS ribosomal sequences between both areas, with one being exclusive of NW Morocco [21]. In section *Acrocentron* and in the *C. toletana* complex, there was also some ITS and ETS ribotypes that were characteristic of Iberian and African species, supporting limitations to gene flow across the Strait of Gibraltar, although their pattern of distribution also showed some contact between species distributed in the different plates [10,19]. Subsequently, in Morocco, and because neopolyploids can be more tolerant to a wider range of environmental conditions, autopolyploid *C. aspera* ssp. *gentilii* may have been better adapted to local habitats and drove their diploid ancestors to extinction [58,59], while in Spain, introgressions among diploid subspecies occurred [46].

Conversely, *C. seridis* showed a high isolation by distance and a high morphological diversity that was correlated with genetic diversity. This species showed a greater genetic structuring and isolation by distance than *C. aspera* in the Iberian Peninsula alone [30]. This could be due to the existence of independent events of allopolyploidization, which was already suggested for *C. seridis* in Spain [30], and/or the fact that it is a highly autogamous species in all its distribution range [35], which may lead to a higher genetic drift in geographically distant populations. *Centaurea seridis* is currently present in both sides of the Strait of Gibraltar, which suggests that it was present in both European and African plates during the Messinian Salinity Crisis, although another hypothesis is a migration mediated by humans. This was the case of *Calicotome villosa* (Poir.) Link, which also showed some isolation by distance, a lack of long-distance dispersal, and a genetic exchange between the African and Iberian sides aided by the movement of cattle and crops [60]. In Morocco, most of the *C. aspera* ssp. *gentilii* and *C. seridis* plants appeared with bites out of them (personal observation). The Moroccan and European individuals were slightly differentiated in the analysis of genomic relationships, although not in the STRUCTURE analysis. This slight differentiation may be due to the recurrent formation of *C. seridis* from differentiated *C. aspera* populations or to divergent evolution given the current parapatric distribution of the species in Morocco and Europe. In addition, small amounts of introgressions from *C. aspera* that represent less than 5% of the genome were observed in Spain. These introgressions were geographically differentiated, the Andalusian *C. seridis* ssp. *sonchifolia* showed some introgressions from the tetraploid *C. aspera* ssp. *gentilii*, and ssp. *maritima*, distributed northernmost, showed some introgressions from diploid *C. aspera*. These introgressions may be due to infrequent hybridizations. In Morocco, *C. seridis* and *C. aspera* ssp. *gentilii* have more parapatric distributions [35], and no introgressions were found. Introgressions with local species are not rare in *Centaurea*. For example, some populations of the autohexaploid *Centaurea amblensis* Graells ssp. *tendudaica* (Rivas Goday) Rivas Mart. showed some introgressions from the diploid *C. ornata* Willd. after polyploidization [17]. Given the geographic (especially latitudinal) pattern of introgressions in *C. seridis* along with a high morphological diversity, they are likely to be adaptive, resulting in phenotypic innovations and local adaptation to geographically distributed conditions [47]. In the same direction, Arnold and Kunte [61] reported that, although genetic exchange may produce maladaptive consequences, a low rate of hybridization, coupled with an opportunity for recombination and selection, will lead to subsequent incorporation of adaptive traits while purging maladaptive traits in the recipient genomes.

In conclusion, our results show that current reproductive interspecific barriers in the *C. aspera* complex, belonging to section *Seridia*, are much stronger than in other *Centaurea* sections. In contrast, polyploidization occurred at least twice, giving rise to the cryptic autopolyploid *C. aspera* ssp. *gentilii* (here, *C. gentilii* is proposed) and the allopolyploid *C. seridis*, which are probably better adapted to the drier Moroccan environment. Within *C. aspera*, a high subspecies structuring is present in Andalusia, close to the Strait of Gibraltar, which appears to be a melting pot of new forms that may promote diversification.

## 4. Materials and Methods

### 4.1. Plant Material

**Figure 6:**
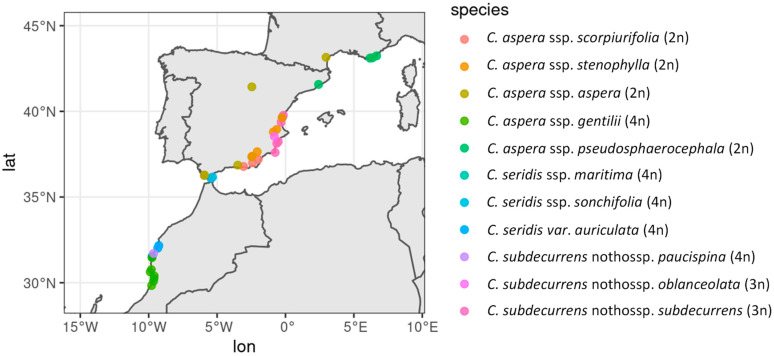
Location of the studied individuals. Map was made with Natural Earth (https://www.naturalearthdata.com/ accessed on 1 June 2022).

Plant sampling included a total of 63 individuals, representing the diversity within *C. aspera* (ssp. *aspera*, ssp. *stenophylla*, ssp. *scorpiurifolia*, ssp. *pseudosphaerocephala*, and ssp. *gentilii*), within *C. seridis* (ssp. *maritima*, ssp. *sonchifolia*, ssp. *cruenta* and var. *auriculata*), and the interspecific hybrids species (triploid *C.* x *subdecurrens* nothossp. *subdecurrens* and nothossp. *oblanceolata*, and tetraploid nothossp. *paucispina*) (Table 4). The studied individuals were distributed from France to Morocco, mainly along the European Mediterranean coast and the Moroccan Atlantic coast (Figure 6).

Plant material was collected from different sampling trips performed in spring. We sampled the Spanish Mediterranean coast in 2009, France in 2010, South Spain in 2010 and 2015, and Morocco in 2012 and 2016. The geographical coordinates of all the sampled individuals were recorded using GPS (Garmin eTrex Vista HCx, Olathe, KS, USA). In all the trips and for each individual, we collected young leaves that were transported in a cooler. A part of them was used immediately for ploidy level analysis, and the other part was stored at −80 °C. Most of the included individuals have already been morphologically characterized, their ploidy level analysed and taxonomically adscribed previously (Table 4) [30,31,46]. For the individuals that are used here for the first time, especially *C. aspera* ssp. *pseudosphaerocephala* and ssp. *gentilii*, and *C. seridis* ssp. *sonchifolia* and var. *auriculata*, ploidy level was analysed from fresh leaf tissue using flow cytometry as described in Garmendia et al. [31]. Prior to the ploidy level screening, initial analyses on some individual plants representing all the diploid subspecies of *C. aspera* and *C. seridis* ssp. *maritima* and var. *auriculata* involving both chromosomes count and flow cytometry were carried out. We used the diploid *C. aspera* ssp. *stenophylla* as a standard control in each individual flow cytometry (Figure S3). The taxonomical adscription of all studied individuals was unambiguously established using general and local floras. Voucher specimens of one representative of each studied taxa are kept in the Herbarium of the Universitat Politècnica de València (VALA) (Table 4).

### 4.2. GBS

Genomic DNA isolation of the sampled individuals was performed following Ferriol et al. [30] shortly after collecting them in the field. The diluted DNA was kept since then at −40 °C. The quality and quantity of the DNA were evaluated immediately before the GBS analysis using spectrophotometry (NanoDrop 2000c, Thermo Scientific, Waltham, MA, USA) and fluorimetry (Qubit 2.0). Isolated genomic DNA was sent to the CNAG-CRG Sequencing Unit in Barcelona (Spain), where GBS was performed and SNPs were identified and selected.

The protocol from Elshire et al. [62] for preparing GBS paired-end multiplexed libraries was implemented and modified with improvements from Poland et al. [63] and Sonah et al. [64]. In brief, due to the limited previous experience with the *Centaurea* genus, a small-scale experiment was performed to select one of the two restriction enzymes (REs) to be used in the future large-scale experiment: ApeKI and PstI. These two REs represent two different restriction patterns at the same sequencing cost: ApeKI captures more of the genome at a lower depth, and PstI captures less of the genome at a higher depth. In the small-scale protocol, the genomic DNA was quantified by Quant-iT™ DNA High-Sensitivity Assay (Thermo Fisher Scientific), and 100 ng/sample was used per reaction. In this pilot phase, four samples were selected, and each sample was digested in two parallel and independent reactions with ApeKI (New England Biolabs, Ipswich, MA, USA) at 75 °C for 2 h and PstI (New England Biolabs) at 37 °C for 2 h. Adaptors compatible with Illumina sequencing were ligated using T4 DNA Ligase (New England Biolabs) to the three or four nucleotide overhangs left after the RE digestion. A titration to determine the adequate adaptor concentration was performed in an independent experiment. Two different adaptor types combined together were used—the “indexed GBS adaptor” and the “common GBS adaptor”—each adaptor mix being specific to the RE used. After the AMPure XP beads (Agencourt, Beckman Coulter, Pasadena, CA, USA) purification, the adaptor ligated reduced representation of the genome. All four samples/RE were pooled, and the pool was PCR amplified with 2x KAPA HiFi HS RM (Roche, Basel, Switzerland). The PCR primers were common to both RE-specific GBS adaptors; one primer was the general primer and the other primer integrated one of eight Illumina barcodes. After the AMPure XP beads purification, the distribution of the fragment sizes within the pool of the sequencing libraries was assessed with an Agilent 2100 Bioanalyzer DNA 7500 assay (Agilent, Santa Clara, CA, USA). The four sequencing libraries corresponding to each RE protocol were sequenced on a MiSeq sequencer (Illumina, San Diego, CA, USA) using a MiSeq v2 flow cell (Illumina) in paired end mode and with a read length of 2 × 151 bp. After this previous analysis, the protocol using the ApeKI RE was selected for a large-scale reduced representation genome sequencing. The large-scale GBS library preparation of the 63 samples was processed within a 96-well plate following the ApeKI RE protocol as described above. The pool of the adaptor ligated libraries consisted of up to 12 individuals and, after the PCR amplification all 63 individuals, were joint into the final sequencing pool. The library pool was sequenced on an Illumina HiSeq2500 (Illumina) in paired-end mode with a read length of 2 × 151 bp using the HiSeq Rapid SBS Kit, following manufacturer’s protocol. Image analysis, base calling and quality scoring of the run were processed using the manufacturer’s software Real Time Analysis (RTA 1.18.66.3) and followed by generation of FASTQ sequence files by CASAVA.

### 4.3. Identification and Selection of SNPs

Passing Illumina’s filters sequences were parsed based on the presence of the enzyme remnant cut site and in-line barcodes with GBS-SNP-CROP [65], and trimming based on quality and adapters were performed with GBS-SNP-CROP and Trimmomatic [66]. These parsed and quality-filtered reads were demultiplexed according to their in-line barcode, and a pair of FASTQ files were produced for each sample with GBS-SNP-CROP. A mock reference was built using a de novo assembly method based on sequence similarity using Pear [67] and Vsearch [68]. Reads were aligned against this reference using BWA-mem [69], and mapped reads were filtered with SAMtools [70]. The properly paired, primary aligned reads were kept to produce an mpileup file for each sample. Variant calling was performed using the GBS-SNP-CROP pipeline, including a series of filters: similarity threshold of 90% for read clustering; selection of the reads which were primary alignment and properly paired; alternate allele strength value of 0.9 (across the population for a given putative bi-allelic variant, this parameter that is the minimum proportion of nonprimary allele reads that are the secondary allele); minimum required ratio of less frequent allele depth to more frequent allele depth of 0.10; minimum acceptable proportion of genotyped individuals to retain a variant of 0.20; maximum missing data proportion of a variant of 0.55; minimum average depth of an acceptable variant of five; and elimination of indels. Subsequently, a genotype matrix (SNP) was generated and converted into a multisample VCF file. A total of 1688 variants which were polymorphic and biallelic were retained for subsequent analyses. Raw data are included in variant call format in Supplementary Data S1.

### 4.4. Genetic Statistical Analysis

The obtained VCF file was read and analysed using vcfR [71] in R [72]. This package was also used to export the SNP data into different formats.

The joint analysis of taxa of different ploidy can be problematic, and part of the available software is only suited for diploid or tetraploid data sets. For the analysis of genetic structure in mixed-ploidy populations, it has been shown that STRUCTURE [73] is more robust than other clustering methods, especially when using large numbers of loci (>1000) [74]. Therefore, we used software STRUCTURE v2.3.4 to estimate the genetic population structure of the sampled *Centaurea* individuals. The admixture model and the correlated allele frequencies between populations options were selected. To estimate the number of populations (*K*), we run STRUCTURE with varying *K* values, ranging from one to nine. Each run consisted of one million burnin iterations and 500,000 data collection iterations and resulted in an estimate of the probability of the data given *K* [ln Pr(*X*/*K*)]. Each value of *K* was evaluated using ten independent Markov chain Monte Carlo replicates. The number of clusters was inferred following Evanno et al. [37], based on the values of Delta *K* for each value of *K* (except for *K* = 1 and the maximum *K* tested).

Subsequent population structure analyses were performed using the R package StaMPP [75], which was specifically designed to calculate genetic differentiation and structure of mixed-ploidy level populations with large data sets such as SNPs. Here, we were not able to precise the exact allele dosage of tetraploids. Consequently, data were recoded as diploid and used within StAMPP to produce results that are less accurate but still biologically meaningful [75]. A genomic relationship matrix which estimates the true proportion of the genome shared between individuals was calculated following Yang et al. [38] and visualized as a heatmap using the R package gplots.

For each cluster obtained using STRUCTURE, SNP variants were used to calculate some measures of population diversity and differentiation using vcfR package. Population mean heterozygosities, G_ST_ [76,77], and G’_ST_ [39] were estimated. Here, G’_ST_ was calculated following Hedrick [39] with the exception that the heterozygosities were weighted by the number of alleles observed in each population to correct for both unbalanced samples and instances where individuals vary in copy number as well, making it appropriate when there is a mixture of ploidies in the sample [78]. We also used the StAMPP package to estimate pairwise fixation index F_ST_, which has been shown to be the better choice for calculating the degree of differentiation between cytotypes [79]. F_ST_ values were estimated along with confidence intervals (5% and 95%) and *p* values between populations by performing 100 bootstrappings across loci and according to the method proposed by Wright [80] and updated by Weir and Cockerham [81]. A hierarchical partition of the genetic variation among species and among the genetic populations found in STRUCTURE was determined with analyses of molecular variance (AMOVAs) using StAMPP based on distances [82] among individuals. As AMOVA is not able to detect admixed populations in contrast with STRUCTURE, all the individuals of the hybrid *C.* x *subdecurrens* were removed from the analysis, and each remaining individual was assigned to a single cluster according to its highest *q_K_*. To test for genetic isolation by distance, the matrix of the previously mentioned genetic distances [82] among individuals obtained with StAMPP was compared with the matrix of geographic distances. We performed a Mantel test [40] using ade4 [83] in R for the clusters obtained with STRUCTURE.

### 4.5. Morphological Characterization

A morphological characterization of the sampled individuals was performed to reveal morphological patterns among the clusters obtained in STRUCTURE. In spring, plants have caulinar leaves, stems, and capitula but lack basal leaves that are already dry. Morphological evaluation was accomplished in the field during the field trips. Table 4 shows the list of the characters that include those traditionally used for differentiation of *Centaurea* section *Seridia* as can be found in determination keys and floras. A total of 38 quantitative variables were evaluated: nine corresponded to reproductive traits and 29 to vegetative traits.

Statistical analysis of morphological data was carried out in R using the packages mass [84] and agricolae [85]. Mean and standard deviation for each quantitative variable and each cluster obtained with STRUCTURE were computed. ANOVAs and post hoc Tukey HSD comparisons among genetic clusters were calculated for all the variables. Bonferroni correction was applied to the ANOVAs significance to correct the effect of several repeated analyses. For subsequent multivariate analysis, twenty vegetative and seven reproductive variables that showed significant differences (*p* < 0.01) among clusters were selected (Table 3). Three principal component analyses (PCA) were performed: with vegetative traits only, with reproductive traits only, and with all the traits together. The matrices of the Nei [80] genetic distances and the Euclidean distances using standardized morphological variables among individuals were compared using a Mantel test as previously described.

## Figures and Tables

**Table 1:** Measures of population diversity and differentiation in *Centaurea aspera* and *C. seridis* using a large SNP set. STRUCTURE analysis was used for establishing genetic populations.

Population	H_S_	G_ST_	G’_ST_	F_ST_		
				F_ST_	5–95% Confidence Interval	*p* Value
*K* = 2						
*C. aspera* (2n and 4n)	0.192	0.209	0.315	0.209	0.196 0.218	0
*C. seridis* (4n)	0.231					
*C.* x *subdecurrens* hybrids (3n and 4n)	0.334					
*K* = 3						
*C. aspera* (2n)	0.165	0.310	0.412	0.279	0.258 0.300	0
*C. aspera* ssp *gentilii* (4n)	0.109					
*C. seridis* (4n)	0.231					
*C.* x *subdecurrens* nothossp. *subdecurrens* and *oblanceolata* (3n)	0.300					
*C.* x *subdecurrens* nothossp. *paucispina* (4n)	0.288					

H_S_: average population heterozygosity, G_ST_: genetic population differentiation, G’_ST_: genetic population differentiation weighted by the number of alleles observed in each population, F_ST_: fixation index, with values along with confidence intervals (5% and 95%) and *p* values between populations following 100 bootstrappings across loci.

**Table 2:** Analyses of molecular variance (AMOVAs) in *Centaurea aspera* and *C. seridis* using a large SNP set. STRUCTURE analysis was used for establishing genetic populations.

Grouping	Source of Variation	d.f.	SSD	MSD	Variance Component	Variance (%)
*Centaurea* species (*C. aspera* and *C. seridis*)	Among species Within ssp.	1 50	0.151 0.545	0.151 0.008	0.007 0.008	46.01 53.98
*Centaurea aspera* 2n and 4n	Among groups Within groups	1 35	0.088 0.296	0.088 0.008	0.005 0.008	39.19 60.81
diploid *Centaurea aspera* cluster 3 and 4	Among groups Within groups	1 25	0.111 0.287	0.111 0.011	0.011 0.011	48.00 52.00

d.f.: degrees of freedom, SSD: sum of squared deviations, MSD: mean squared deviations.

**Table 3:** ANOVAs of the morphological characters among the *Centaurea* clusters obtained using STRUCTURE (twenty-six diploid *C. aspera* individuals, ten tetraploid *C. aspera* ssp. *gentilii*, fifteen tetraploid *C. seridis*, six triploid *C.* x *subdecurrens* (nothossp. *subdecurrens* and nothossp. *oblanceolata*), and five tetraploid *C.* x *subdecurrens* nothossp. *paucispina* individuals). Bonferroni correction, mean and standard error are indicated. Different letters indicate differences among clusters resulting from post hoc Tukey HSD tests (THDS). *: variables used for PCA.

Variable	*p* (ANOVA)	*p* (Bonf. Corr.)	*C. aspera* (2n)		*C. aspera* ssp. *gentilii* (4n)		*C. seridis* (4n)		*C.* x *subdecurrens* nothossp. *subdecurrens* and *oblanceolata* (3n)		*C.* x *subdecurrens* nothossp. *paucispina* (4n)	
			Mean ± st. Error	THDS	Mean ± st. Error	THDS	Mean ± st. Error	THDS	Mean ± st. Error	THDS	Mean ± st. Error	THDS
	Vegetative characters											
Plant height (cm)	0.010	0.302	50.23 ± 4.70	a	29.50 ± 6.86	a	34.33 ± 7.45	a	57.83 ± 6.92	a	21.00 ± 2.45	a
Maximum plant diameter (cm)	0.150	1.000	105.08 ± 10.30	a	79.70 ± 20.23	a	114.47 ± 15.75	a	144.00 ± 23.18	a	73.00 ± 14.63	a
Perpendicular plant diameter (cm)	0.055	1.000	84.38 ± 8.78	a	58.00 ± 12.30	a	91.67 ± 13.15	a	122.33 ± 30.06	a	50.00 ± 7.25	a
Plant volume (dm^3^)	0.095	1.000	637.51 ± 135.00	a	363.75 ± 255.85	a	649.48 ± 262.85	a	1420.428 ± 528.67	a	87.45 ± 28.61	a
Stem diameter (mm) *	<0.001	<0.001	3.07 ± 0.26	b	3.26 ± 0.22	b	5.22 ± 0.29	a	4.39 ± 0.49	ab	3.52 ± 0.38	b
Upper leaf: internode length (mm)	0.002	0.058	11.52 ± 1.28	ab	7.04 ± 1.12	b	14.64 ± 2.28	ab	19.49 ± 2.26	a	6.02 ± 1.50	b
Upper leaf: decurrence length (mm) *	<0.001	0.001	0.00 ± 0.00	b	0.00 ± 0.00	b	10.19 ± 3.04	a	4.63 ± 0.90	ab	0.96 ± 0.96	b
Upper leaf: decurrence proportion *	<0.001	<0.001	0.00 ± 0.00	b	0.00 ± 0.00	b	0.53 ± 0.12	a	0.26 ± 0.04	ab	0.08 ± 0.08	b
Upper leaf: decurrence width (mm) *	<0.001	<0.001	0.00 ± 0.00	b	0.00 ± 0.00	b	3.50 ± 0.83	a	2.14 ± 0.23	ab	0.86 ± 0.86	b
Upper leaf: total length (mm) *	<0.001	<0.001	21.56 ± 1.35	c	21.62 ± 1.87	c	41.81 ± 4.63	a	24.41 ± 2.08	bc	40.76 ± 2195	ab
Upper leaf: total width (mm) *	<0.001	<0.001	4.28 ± 0.49	b	5.87 ± 0.70	b	17.31 ± 2.30	a	5.39 ± 0.91	b	9.00 ± 0.85	b
Upper leaf: roundness *	<0.001	<0.001	0.19 ± 0.02	b	0.28 ± 0.03	b	0.41 ± 0.03	a	0.22 ± 0.02	b	0.22 ± 0.02	b
Upper leaf: thickness without vein (mm) *	<0.001	<0.001	0.28 ± 0.02	b	0.45 ± 0.03	a	0.55 ± 0.05	a	0.44 ± 0.02	a	0.41 ± 0.03	ab
Upper leaf: thickness with vein *	<0.001	<0.001	0.42 ± 0.03	c	0.55 ± 0.04	bc	0.91 ± 0.07	a	0.63 ± 0.06	bc	0.69 ± 0.05	ab
Upper leaf: apical lobe length (mm) *	<0.001	<0.001	19.88 ± 1.45	b	21.62 ± 1.87	b	36.52 ± 3.79	a	22.63 ± 2.28	b	40.76 ± 2.19	a
Upper leaf: apical lobe width (mm) *	<0.001	<0.001	3.87 ± 0.45	b	5.87 ± 0.70	b	16.22 ± 1.86	a	5.22 ± 0.92	b	9.00 ± 0.85	b
Upper leaf: number of lobes	0.478	1.00	1.40 ± 0.24	a	1.00 ± 0.00	a	1.87 ± 0.48	a	1.33 ± 0.33	a	1.00 ± 0.00	a
Medium leaf: internode length (mm) *	0.001	0.026	25.66 ± 2.47	a	11.43 ± 2.19	b	33.84 ± 3.82	a	30.46 ± 4.16	a	23.19 ± 4.49	ab
Medium leaf: decurrence length (mm) *	<0.001	<0.001	0.00 ± 0.00	b	0.00 ± 0.00	b	24.47 ± 6.58	a	12.22 ± 2.32	ab	4.96 ± 1.32	b
Medium leaf: decurrence proportion *	<0.001	<0.001	0.00 ± 0.00	b	0.00 ± 0.00	b	0.70 ± 0.17	a	0.42 ± 0.09	ab	0.21 ± 0.06	ab
Medium leaf: decurrence width (mm) *	<0.001	<0.001	0.00 ± 0.00	c	0.00 ± 0.00	c	7.22 ± 0.66	a	5.24 ± 0.64	ab	4.03 ± 1.29	b
Medium leaf: total length (mm) *	<0.001	<0.001	49.46 ± 2.89	c	60.31 ± 6.01	bc	91.00 ± 5.60	a	74.22 ± 5.85	ab	58.87 ± 3.37	bc
Medium leaf: total width (mm) *	<0.001	<0.001	14.92 ± 1.61	b	19.52 ± 2.72	b	36.93 ± 3.18	a	23.64 ± 1.71	b	21.90 ± 3.09	b
Medium leaf: roundness	0.012	0.351	0.29 ± 0.02	b	0.33 ± 0.03	ab	0.42 ± 0.03	a	0.32 ± 0.01	ab	0.36 ± 0.03	ab
Medium leaf: thickness without vein (mm) *	<0.001	<0.001	0.27 ± 0.02	b	0.43 ± 0.04	a	0.57 ± 0.05	a	0.40 ± 0.03	ab	0.47 ± 0.04	a
Medium leaf: thickness with vein *	<0.001	<0.001	0.62 ± 0.04	c	0.96 ± 0.09	b	1.31 ± 0.09	a	0.80 ± 0.09	bc	1.19 ± 0.11	ab
Medium leaf: apical lobe length (mm)	0.100	1.00	30.72 ± 3.08	a	37.85 ± 7.58	a	45.56 ± 5.43	a	35.94 ± 9.85	a	22.90 ± 3.18	a
Medium leaf: apical lobe width (mm) *	<0.001	<0.001	11.58 ± 1.76	b	16.66 ± 3.05	b	31.47 ± 2.47	a	14.23 ± 2.62	b	16.22 ± 3.29	b
Medium leaf: number of lobes	0.055	1.00	4.10 ± 0.54	a	3.70 ± 1.06	a	5.87 ± 0.73	a	7.83 ± 2.33	a	4.50 ± 0.22	a
	Reproductive characters											
Number of capitula per branch	0.024	0.218	16.71 ± 4.00	a	4.25 ± 1.70	a	10.43 ± 3.65	a	27.08 ± 6.65	a	0.80 ± 0.12	a
Capitulum length (mm) *	<0.001	<0.001	25.89 ± 0.63	c	28.53 ± 1.73	bc	36.57 ± 1.23	a	31.48 ± 0.35	ab	34.38 ± 1.93	ab
Involucre length (mm) *	<0.001	<0.001	12.77 ± 0.30	d	13.44 ± 0.66	cd	18.51 ± 0.41	a	15.81 ± 0.41	bc	17.58 ± 0.89	ab
Involucre width (mm) *	<0.001	<0.001	9.03 ± 0.48	b	7.88 ± 0.63	b	15.33 ± 0.98	a	11.18 ± 0.39	b	10.78 ± 0.71	b
Roundness of involucre *	0.003	0.026	0.70 ± 0.03	ab	0.58 ± 0.02	b	0.83 ± 0.06	a	0.71 ± 0.03	ab	0.61 ± 0.10	ab
Length of longest bract spine (mm) *	<0.001	<0.001	2.65 ± 0.27	bc	1.46 ± 0.20	c	7.44 ± 0.46	a	3.50 ± 0.52	b	3.32 ± 0.28	bc
Number of spines per bract *	<0.001	<0.001	4.92 ± 0.26	c	3.89 ± 0.35	c	7.77 ± 0.37	a	9.67 ± 0.33	ab	5.40 ± 0.40	bc
Number of outer flowers per capitulum	0.285	1.000	13.06 ± 1.21	a	10.28 ± 0.73	a	14.29 ± 0.44	a	13.67 ± 0.87	a	13.40 ± 0.51	a
Number of inner flowers per capitulum *	<0.001	<0.001	22.83 ± 2.25	b	15.94 ± 1.77	b	37.39 ± 2.59	a	25.83 ± 3.43	ab	27.60 ± 2.14	ab

**Table 4:** Taxonomic adscription, location, and voucher number if available for all the studied *Centaurea* individuals. * Individuals that have already been morphologically characterized, their ploidy level analysed and taxonomically adscribed previously.

Species	Infraspecific Adscription Ploidy Level/Voucher Number	Country	Locality	Geographic Coordinates
*Centaurea aspera*	ssp. *aspera* 2n VALA 9604	Spain	Los Porteros	N37 20.909 W2 27.727
			Almazán *	N41 26.013 W2 27.332
			Libreros *	N36 17.619 W5 55.314
			Vejer de la Frontera *	N36 15.217 W5 56.368
			Vélez de Benaudalla *	N36 51.660 W3 29.205
		France	Narbonne	N43 09.248 E2 57.806
	ssp. *stenophylla* 2n VALA 9495	Spain	Santa Pola *	N38 14.175 W0 31.270
			Calblanque *	N37 36.037 W0 45.095
			Chilches *	N39 46.445 W0 9.144
			Fuente la Higuera	N38 47.775 W0 53.155
			Guardamar del Segura *	N38 07.678 W0 38.537
			Marjal dels Moros *	N39 37.385 W0 15.178
			Marjal dels Moros *	N39 37.362 W0 15.217
			Montesa	N38 56.698 W0 38.613
			Sax *	N38 32.472 W0 48.990
			Vélez-Rubio *	N37 38.635 W2 04.047
			Tíjola *	N37 22.747 W2 26.583
			El Saler *	N39 22.220 W 0 19.275
	ssp. *scorpiurifolia* 2n VALA 9582	Spain	Bédar *	N37 11.950 W1 59.729
			Bédar *	N37 11.967 W1 59.707
			Alhamilla *	N36 59.614 W2 24.619
			Alhamilla *	N36 59.615 W2 24.636
			La Parra *	N36 46.970 W3 03.969
	ssp. *pseudosphaerocephala* 2n VALA 9615	France	Les Hyères	N43 06.449 E6 10.826
			Le Lavandou	N43 07.512 E6 21.787
			Pampelonne	N43 14.301 E6 39.782
		Spain	Mataró	N41 34.371 E2 25.191
	ssp. *gentilii* 4n VALA 9623	Morocco	Sidi R’bat	N30 4.890 W9 38.806
			Timzilt	N29 49.948 W9 47.526
			Timzilt	N29 49.949 W9 47.526
			Agadir	N30 23.926 W9 35.797
			Cap Ghir	N30 37.695 W9 53.251
			Essaouira (South)	N31 27.813 W9 45.389
			Tamri	N30 45.808 W9 49.342
			Zaouiat El Kourati *	N31 42.815 W9 38.388
			Zaouiat El Kourati *	N31 42.857 W9 38.442
			Takate	N30 15.415 W9 36.377
*Centaurea seridis*	ssp. *maritima* 4n VALA 9496	Spain	Canet *	N39 40.760 W0 12.188
			Calblanque *	N37 36.140 W0 43.940
			Chilches *	N39 46.470 W0 9.0.024
			Santa Pola *	N38 14.203 W0 31.933
			El Saler *	N39 22.218 W0 19.303
	ssp. *sonchifolia* 4n/VALA 9621	Spain	La Línea de la Concepción	N36 9.650 W5 20.334
			Algeciras	N36 5.713 W5 26.688
	ssp. *cruenta* 4n/VALA 9508	Spain	Sax *	N38 32.475 W0 48.957
	var. *auriculata* 4n VALA 9624	Morocco	Essaouira (North)	N31 31.699 W9 45.016
			Souira Kedima	N32 1.439 W9 20.0078
			Souira Kedima	N32 1.457 W9 20.0298
			Safi	N32 10.119 W9 15.798
			Safi	N32 10.233 W9 15.712
			Zaouiat El Kourati *	N31 43.163 W9 38.009
			Zaouiat El Kourati *	N31 43.159 W9 38.146
*Centaurea* x *subdecurrens*	nothossp. *subdecurrens* 3n VALA 9500	Spain	Guardamar del Segura *	N38 07.677 W0 38.537
			Santa Pola *	N38 13.875 W0 30.908
			Calblanque *	N37 35.967 W0 45.427
			Chilches *	N39 46.279 W0 9.144
	nothossp. *oblanceolata* 3n/VALA 9509	Spain	Sax *	N38 32.475 W0 48.955
			Sax *	N38 32.465 W0 48.948
	nothossp. *paucispina* 4n VALA 9519	Morocco	Zaouiat El Kourati *	N31 42.859 W9 38.437
			Zaouiat El Kourati *	N31 42.836 W9 38.408
			Zaouiat El Kourati *	N31 42.842 W9 38.408
			Zaouiat El Kourati *	N31 42.815 W9 38.388
			Zaouiat El Kourati *	N31 42.841 W9 38.410

## Data Availability

Dcnagata is contained within the article or supplementary material.

## Supplementary Materials

The following supporting information can be downloaded at: https://www.mdpi.com/article/10.3390/plants11151919/s1, Figure S1: STRUCTURE clustering analysis for *Centaurea aspera*, *C. seridis* and hybrids between them in the studied area for *K* = 4; Figure S2: Two-dimensional plot of the principal component analysis based on the morphology of the *Centaurea aspera* and *C. seridis* individuals. STRUCTURE analysis was used for establishing *K* = 4 genetic populations; Figure S3: Flow cytometry histograms based on DAPI fluorescence staining and analysed with the CyView software (Partec) obtained for different *Centaurea* individuals. Table S1: Measures of population diversity and differentiation in *Centaurea aspera* and *C. seridis* using a large SNP set. STRUCTURE analysis was used for establishing *k* = 4 genetic populations; Data S1: GBS raw data in vcf format.
