# Supplementary material for: Genotyping-by-Sequencing and Morphology Revealed the Role of Polyploidization and Hybridization in the Diversification of the Centaurea aspera L. Complex of Section Seridia (Juss.) DC. (Asteraceae)
Source: Plants (Basel). 2022 Jul 25;11(15):1919. doi: 10.3390/plants11151919 (PMC9332712; doi:10.3390/plants11151919)

Figure S1.

STRUCTURE clustering analysis for *Centaurea aspera*, *C. seridis* and hybrids between them in the studied area for *K* = 4. A. Diagram representing alignment test with individual plants represented by columns and taxonomic adscription of individuals indicated. B. Geographic location of STRUCTURE clusters. Pie charts are depicted in contact zones.

A.

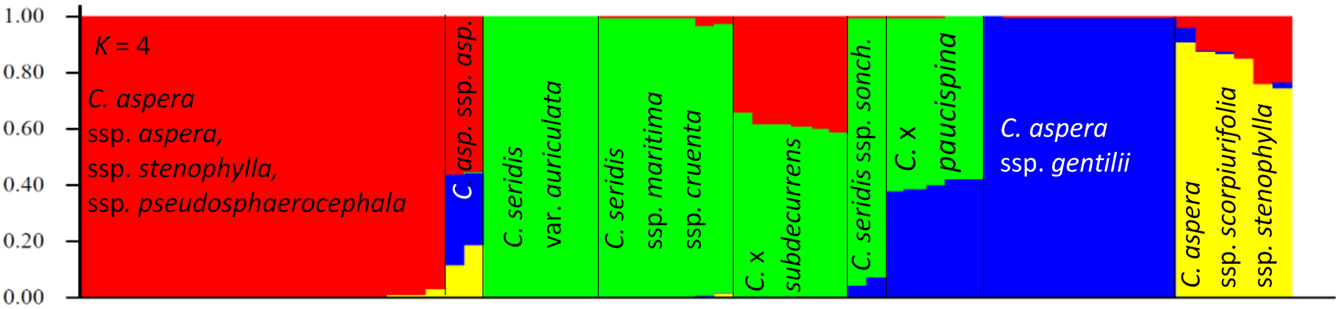

B.

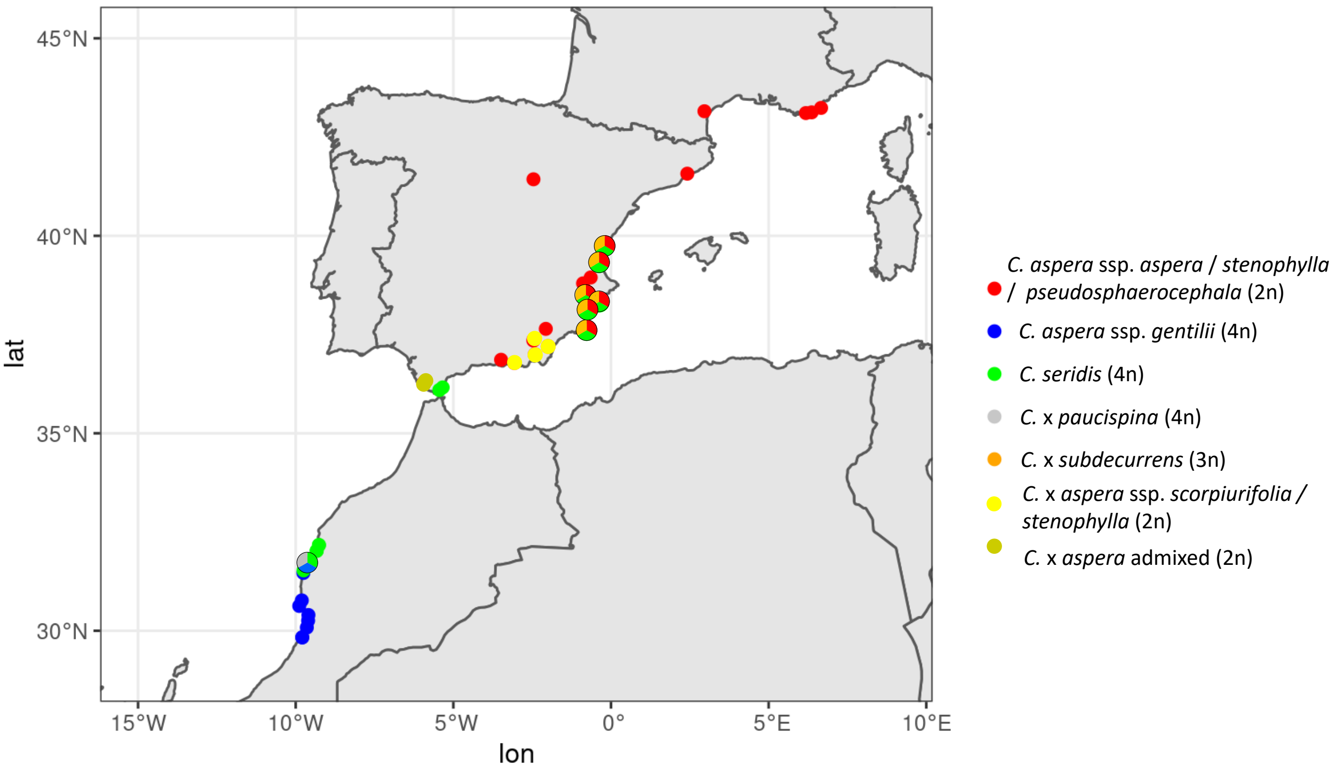

Supplement: Supplementary file 1 [file plants-11-01919-s001.zip › Figure S1.pdf]
