# Supplementary material for: Genotyping-by-Sequencing and Morphology Revealed the Role of Polyploidization and Hybridization in the Diversification of the Centaurea aspera L. Complex of Section Seridia (Juss.) DC. (Asteraceae)
Source: Plants (Basel). 2022 Jul 25;11(15):1919. doi: 10.3390/plants11151919 (PMC9332712; doi:10.3390/plants11151919)

Figure S2.

Two-dimensional plot of the Principal Component Analysis based on the morphology of the *Centaurea aspera* and *C. seridis* individuals. STRUCTURE analysis was used for establishing K = 4 genetic populations. A. Vegetative characters B. Reproductive characters. C. Vegetative and reproductive characters.

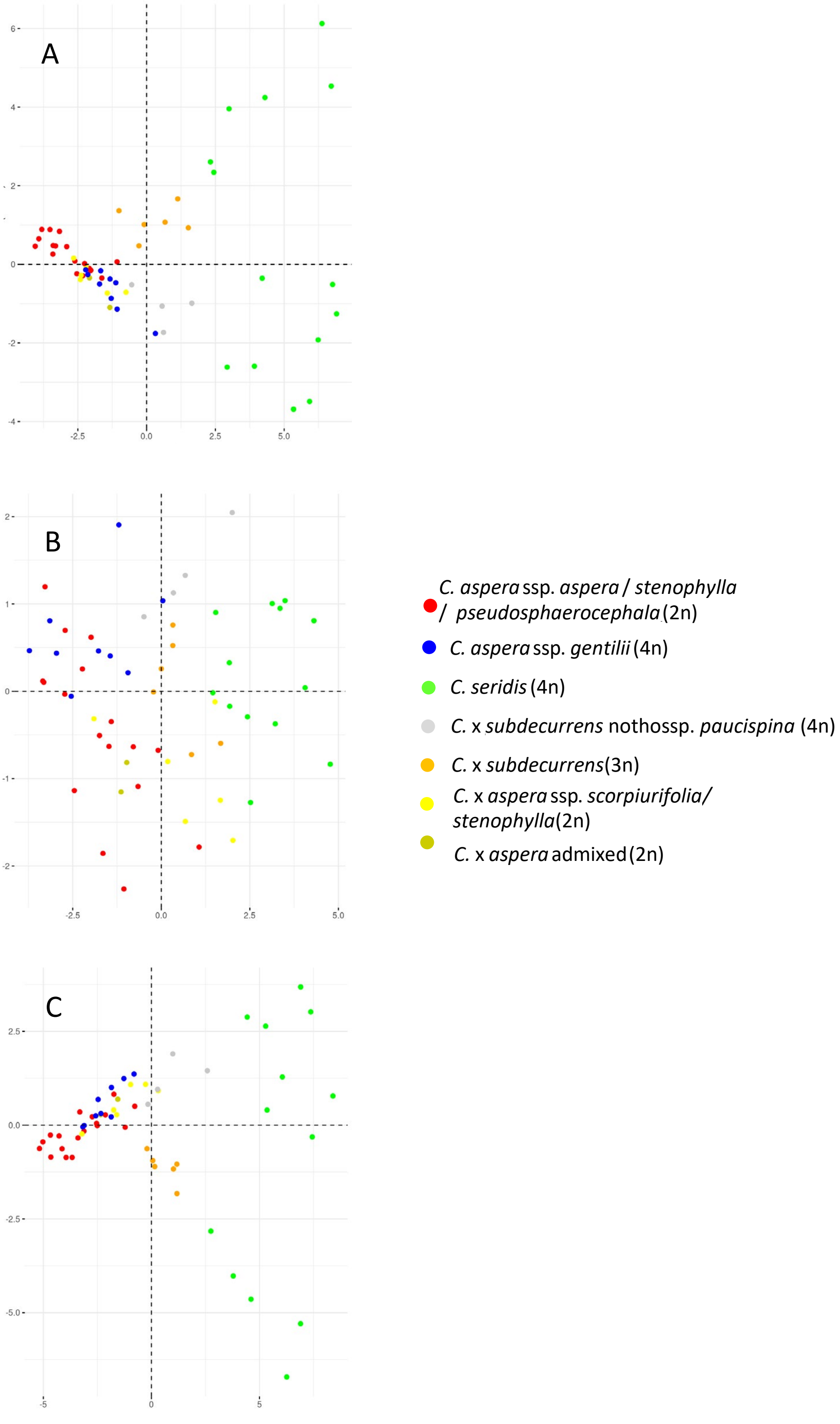

Supplement: Supplementary file 1 [file plants-11-01919-s001.zip › Figure S2.pdf]
