# Supplementary material for: Genotyping-by-Sequencing and Morphology Revealed the Role of Polyploidization and Hybridization in the Diversification of the Centaurea aspera L. Complex of Section Seridia (Juss.) DC. (Asteraceae)
Source: Plants (Basel). 2022 Jul 25;11(15):1919. doi: 10.3390/plants11151919 (PMC9332712; doi:10.3390/plants11151919)
Supplement: Supplementary file 1 [file plants-11-01919-s001.zip › Figure S3.pdf]

Figure S3.

Flow cytometry histograms based on DAPI fluorescence staining and analysed with the CyView software (Partec) obtained for different *Centaurea* individuals. A. Diploid *C. aspera* ssp. *pseudosphaerocephala*; B. Triploid *C.* x *subdecurrens* nothossp. *subdecurrens*; C. Tetraploid *C. seridis* var. *auriculata*. Peak number 1 corresponds to the diploid internal control (*C. aspera* ssp. *stenophylla*). Peak positions are depicted, and number of particles (particle), coefficient of variation (CV), arithmetic mean (center) and median (median) of samples are also included in the tables.

A.

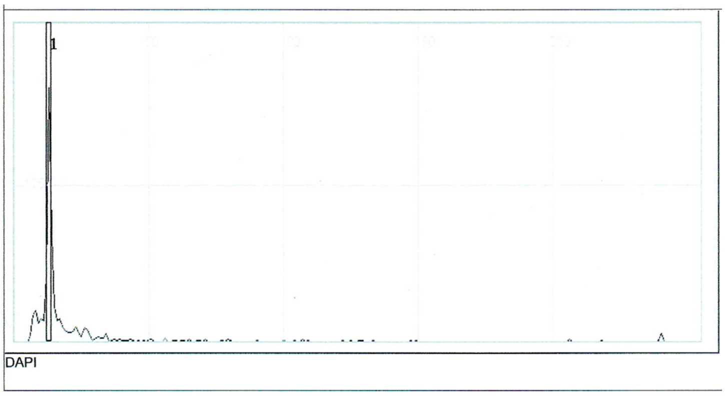


REGIONS

| Name | Particle | Center | CV% | Median |
|---|---|---|---|---|
| DAP_1 | 203 | 13.00 | 6.86 | 13 |

B.

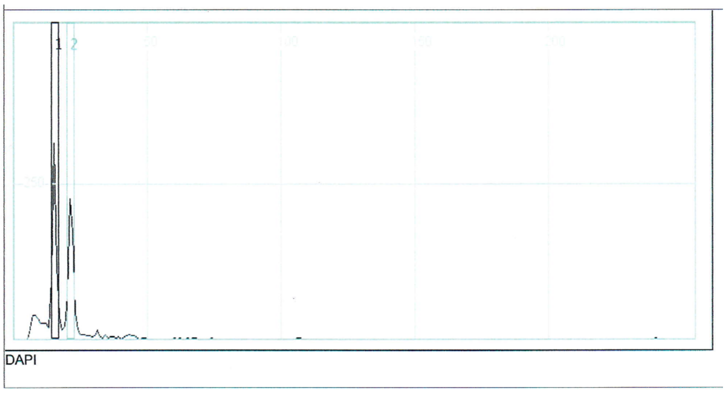


REGIONS

| Name | Particle | Center | CV% | Median |
|---|---|---|---|---|
| DAP_1 | 435 | 15.00 | 4.24 | 15 |
| DAP_2 | 399 | 22.52 | 3.20 | 21 |

C.

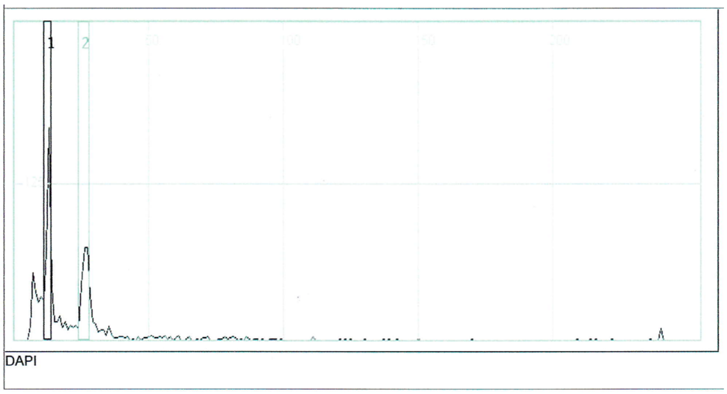


REGIONS

| Name | Particle | Center | CV% | Median |
|---|---|---|---|---|
| DAP_1 | 241 | 13.00 | 6.62 | 13 |
| DAP_2 | 192 | 26.00 | 3.37 | 26 |
