# Supplementary material for: Genotyping-by-Sequencing and Morphology Revealed the Role of Polyploidization and Hybridization in the Diversification of the Centaurea aspera L. Complex of Section Seridia (Juss.) DC. (Asteraceae)
Source: Plants (Basel). 2022 Jul 25;11(15):1919. doi: 10.3390/plants11151919 (PMC9332712; doi:10.3390/plants11151919)
Supplement: Supplementary file 1 [file plants-11-01919-s001.zip › Table S1.pdf]

Table S1.

Measures of population diversity and differentiation in *Centaurea aspera* and *C. seridis* using a large SNP set. STRUCTURE analysis was used for establishing *k* = 4 genetic populations.

| Population | $H_S$ | $H_T$ | n | $G'_{ST}$ | $F_{ST}$ | | |
|---|---|---|---|---|---|---|---|
| | | | | | $F_{ST}$ | 5% - 95% confidence interval | *p*-value |
| *C. aspera* cluster 1 (2n) | 0.144 | 0.291 | 22.047 | 0.461 | 0.271 | 0.242 0.295 | 0 |
| *C. aspera* cluster 2 (2n) | 0.088 | | 6.286 | | | | |
| *C. aspera* spp. *gentilii* (4n) | 0.109 | | 9.966 | | | | |
| *C. seridis* (4n) | 0.231 | | 14.491 | | | | |
| *C.* x *subdecurrens* (3n) | 0.300 | | 5.794 | | | | |
| *C.* x *subdecurrens* nothossp. *paucispina* (4n) | 0.288 | | 4.389 | | | | |

$H_S$: average population heterozygosity, $H_T$: Total population heterozygosity, n: number of alleles observed in each population, $G'_{ST}$ : genetic population differentiation $G'_{ST}$ weighted by the number of alleles observed in each population, $F_{ST}$ : Fixation index, values along with confidence intervals (5% and 95%) and *p*-values between populations following 100 bootstrappings across loci.
